# Remedies Worth Trying in Eczematous Eruptions of Dogs

**Published:** 1898-02

**Authors:** 


### REMEDIES WORTH TRYING IN ECZEMATOUS ERUPTIONS OF DOGS.

Ichthyol ointment, 2 to 5 per cent., with equal parts of lanolin and cosmoline.

Ichthyol tar-soap moistened and rubbed on the itching parts and allowed to dry.

Permanganate of potash solution of the strength of 1 to 3 per cent. where the dogs are peculiarly irritable.

Where the surfaces are widespread and moist, dust with equal parts of talcum, lycopodium, and boric acid.

For use in the house on pet-dogs that have the range of the parlor and lounge on the rugs and furniture, try a 15 per cent. solution of hyposulphite of soda.

Creoline baths of the strength of 5 per cent. will give very great relief for many hours.

In the finer toy varieties, try a 1 per cent. solution of trikresol; ten grains of boric acid to the ounce of this solution adds to its efficacy in some cases.

Chloro-naphtholeum, two ounces to the gallon of water, is a useful medicated bath for the large varieties of dogs, and is destructive to parasitic forms of life.

Trikresol soap is far safer and better than carbolic acid soap for bath purposes when washing dogs frequently.

Do not use bichloride of mercury solutions in moist eczema.

Starch and oxide of zinc may be used separately or in combination as a dusting-powder alone, or after any of the watery solutions, and tend to hasten healing of broken or denuded parts.

In chronic and long-standing cases, where fissures of the skin have formed and the animal tends to bite the affected parts, causing bleeding, touch them a few times with Monsell's solution of iron, and then use one of the powders.

For softening the parts, equal portions of lanolin and pure cosmoline, with ten grains of boric acid, will be found useful and cleansing.

